# Epigenetic Regulation of Production Traits in Ruminants: Implications for Breeding and Selection

**DOI:** 10.3390/biology15050416

**Published:** 2026-03-03

**Authors:** Huaijing Liu, Mewangling Qumu, Ying Lu, Keyu Li, Yuwei Qian, Zhengmei Sheng, Jinpeng Shi, Dongmei Xi, Jiao Wu

**Affiliations:** Yunnan Provincial Key Laboratory of Animal Nutrition and Feed, Faculty of Animal Science and Technology, Yunnan Agricultural University, Kunming 650201, China; 18361309553@163.com (H.L.); 18083829153@163.com (M.Q.); yinglu_1998@163.com (Y.L.); 17787916184@163.com (K.L.); 15368050094@163.com (Y.Q.); 18008847283@163.com (Z.S.); sjp020607@163.com (J.S.)

**Keywords:** epigenetics, ruminant breeding, trait regulation, environmental response, biomarkers

## Abstract

The important economic traits of ruminants result from interactions between genetic background and environmental factors, but key traits such as reproductive performance, feed efficiency, disease resistance, and livestock product quality are often not fully explained by DNA sequence variations alone. Increasing evidence suggests that epigenetic regulation serves as a crucial molecular bridge connecting environmental stimuli with changes in gene expression, allowing organisms to exhibit stable and plastic phenotypic differences without altering the DNA sequence. This review provides a structured synthesis of recent research in the field of epigenetics in ruminants, elucidating how multiple layers of epigenetic mechanisms, including DNA methylation, histone modifications, non-coding RNAs, and higher-order chromatin structures, coordinate to regulate growth, development, reproductive performance, metabolic and immune homeostasis, and livestock product traits across different tissues and developmental stages. These epigenetic marks not only demonstrate high responsiveness to nutrition, management, and environmental stressors, but can exhibit context-dependent stability within the same tissue and physiological stage when environmental conditions are comparable, thereby contributing to the regulation of phenotypic plasticity and offering potential value as predictive biomarkers. Furthermore, epigenetic information can supplement our understanding of phenotypic variation in ways that traditional genomic selection methods are unable to capture, offering new data dimensions for the prediction and improvement of low heritability, environmentally sensitive traits. Overall, integrating epigenetic information with genomic selection strategies may improve the accuracy of ruminant trait prediction and enhance environmental adaptability. This integration also offers a conceptual basis and technical pathway for developing more precise and sustainable breeding systems.

## Simple Summary

Ruminant production depends on complex traits such as growth, fertility, health, and product quality, which are shaped by both genetic background and environmental conditions. Epigenetic regulation links environmental signals to gene activity without altering DNA sequences, thereby influencing trait development. This review summarizes recent advances in epigenetic studies in ruminants and discusses how DNA methylation, histone modifications, non-coding RNAs, and chromatin organization contribute to phenotypic variation. We also evaluate the potential for epigenetic markers to be complementary tools to genomic selection, particularly for traits with low heritability or strong environmental sensitivity. Integrating epigenetic information may support more precise and sustainable breeding strategies in ruminant production.

## 1. Introduction

Ruminants, such as cattle and sheep, supply a major share of global animal protein, with over 50% of livestock-sector protein originating from these species, while also supporting the livelihoods of hundreds of millions of people, particularly in low- and middle-income regions. Their productivity and environmental adaptability directly impact the sustainability of the livestock industry [1]. For a long time, genetic improvements in ruminants have primarily relied on pedigree records and phenotypic measurements, combined with modern breeding methods such as Best Linear Unbiased Prediction (BLUP) and Genomic Selection (GS) [2]. These methods enable precise evaluation of individual breeding values by integrating genome-wide genetic markers, significantly shortening generation intervals and accelerating the rate of genetic progress [3,4]. However, this DNA sequence variation-based breeding paradigm still has inherent limitations. Several key economic traits are difficult or costly to assess in live animals or at early developmental stages. These include feed efficiency (requiring individual feed intake measurements), carcass quality (reliably assessed only post-slaughter), and disease resistance (often dependent on pathogen challenge or specialized immune assays). These traits also tend to exhibit low heritability and strong sensitivity to nutrition, management, and environmental conditions [5,6]. More fundamentally, traditional genetic models cannot fully account for how environmental differences, in the absence of significant DNA sequence variation, translate into markedly different phenotypic outcomes through regulatory mechanisms [7].

Organisms can exhibit sustained and organized transcriptional responses to external environmental stimuli through epigenetic regulatory mechanisms. This phenomenon has significant biological implications in the growth, reproduction, and metabolic regulation of ruminants [8,9]. Studies have demonstrated that epigenetic regulatory mechanisms—including DNA methylation, histone modifications, non-coding RNAs, and chromatin structure—play essential roles in regulating gene expression across different tissues and developmental stages while maintaining a certain degree of stability [10]. These epigenetic modifications not only reflect the ability of individuals to adapt to environmental changes but also influence phenotypic traits in ruminants, including growth, reproduction, and immunity, through their effects on multiple biological processes [11].

With the rapid advancement of high-throughput sequencing and multi-omics technologies, epigenetic research has shifted from exploring individual mechanisms to systemic analyses [12]. The application of technologies such as whole-genome methylation sequencing, chromatin accessibility analysis, histone modification profiling, and non-coding RNA sequencing has uncovered epigenetic variation patterns associated with traits such as growth, reproduction, metabolism, and immunity [13]. The introduction of epigenome-wide association studies (EWASs) and methylation quantitative trait locus (meQTL) analyses has facilitated the transition of epigenetic markers from basic research to biomarker development and trait prediction, providing new data sources and analytical methods for breeding [14,15].

**Figure 1:**
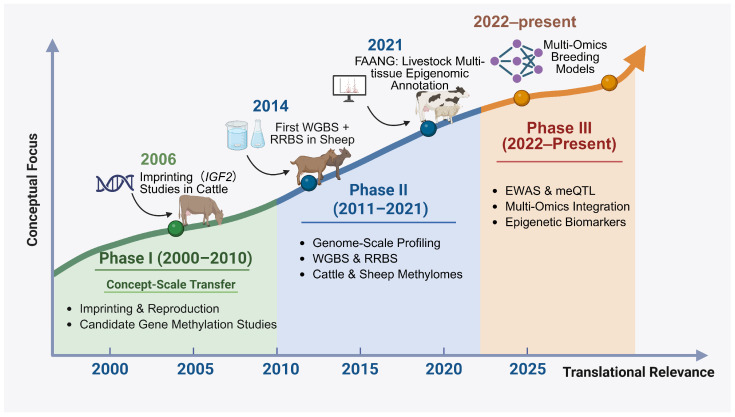
Timeline of the evolution of epigenetic research in ruminants (2000–2025). Representative studies associated with each developmental phase are discussed in the main text. The timeline illustrates the progressive shift in ruminant epigenetic research from early concept-driven and candidate-gene studies (Phase I, 2000–2010), through genome-scale epigenomic profiling (Phase II, 2011–2021), to integrative and translational applications (Phase III, 2022–present). Abbreviations: *IGF2*, Insulin-like growth factor 2; WGBS, Whole-genome bisulfite sequencing; RRBS, Reduced representation bisulfite sequencing; EWAS, Epigenome-wide association study; meQTL, Methylation quantitative trait loci; FAANG, Functional Annotation of Animal Genomes; Multi-Omics, Integrated analysis of genomics, epigenomics, transcriptomics, proteomics, and metabolomics.

Over the past two decades, epigenetic research in ruminants has evolved through several distinct developmental stages. Early studies around 2006 focused primarily on imprinting and candidate-gene methylation analyses, exemplified by investigations of *IGF2* imprinting and reproduction-related epigenetic regulation in cattle [16]. Around 2014, the first applications of genome-scale approaches such as whole-genome bisulfite sequencing (WGBS) and reduced representation bisulfite sequencing (RRBS) in sheep marked a transition toward high-resolution methylome profiling [17]. The launch and expansion of the Functional Annotation of Animal Genomes (FAANG) initiative in 2021 further accelerated livestock multi-tissue epigenomic mapping and integrative functional annotation efforts [18]. Since 2022, research has increasingly shifted toward translational applications, including EWASs, meQTL analyses, and multi-omics integration for trait prediction and breeding-relevant biomarker development [9]. To provide a structured overview of this developmental trajectory, Figure 1 summarizes the major milestones and conceptual shifts in ruminant epigenetic research from 2000 to the present.

This review presents a structured narrative synthesis of recent advances in ruminant epigenetic research, drawing on literature identified through searches of major scientific databases using keywords related to ruminant and epigenetic regulation. The review focuses on how epigenetic regulation contributes to trait formation across different tissues, developmental stages, and major production-related phenotypes, including growth, reproduction, metabolism, immunity, and livestock product quality. Particular attention is given to studies in which epigenetic variation is directly linked to phenotypic differences, trait prediction, or breeding-relevant outcomes, rather than on descriptive epigenetic profiling alone. In addition, we discuss how epigenetic mechanisms mediate the effects of nutrition, management, and environmental stress, providing biological insight into phenotypic variation and genotype–environment interactions not fully explained by DNA sequence variation. Through this integrative synthesis, we aim to clarify the role of epigenetic regulation in shaping complex traits in ruminants and summarize current evidence supporting its relevance to breeding and production practice. To assist non-specialist readers, a glossary of key epigenetic terms is provided in Supplementary Table S5.

## 2. Overview of Epigenetic Regulatory Mechanisms

**Figure 2:**
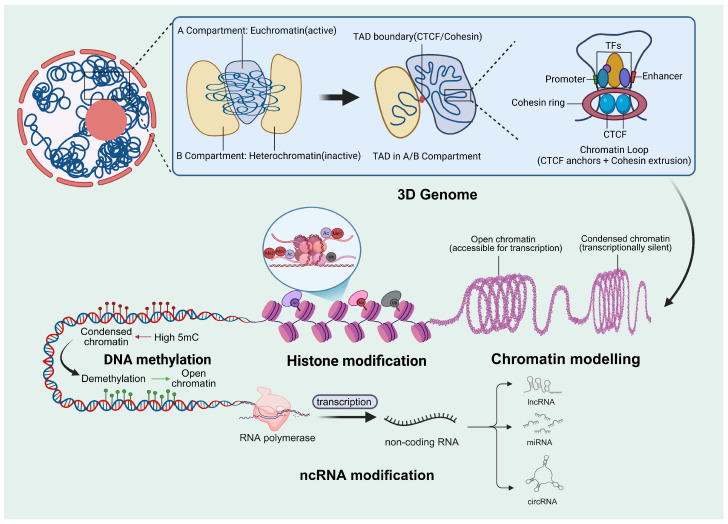
Integrated layers of epigenetic regulation shaping gene expression. For clarity, the main figure presents a conceptual overview of epigenetic regulatory layers, whereas additional mechanistic details are provided in Supplementary Figure S1. The diagram summarizes epigenetic regulation across spatial scales. At the 3D genome architecture level, chromosomes segregate into active A (euchromatin) and inactive B (heterochromatin) compartments containing TADs. TAD boundaries are enriched for CTCF and cohesin, which mediate loop extrusion and promoter-enhancer contacts. Chromatin states range from open (transcription-permissive) to condensed (transcriptionally silent). Histone modifications include activating marks such as Ac and H3K4me3, and repressive marks such as H3K27me3, as well as mono-, di-, and tri-methylation (Me1/2/3) and Ub. DNA methylation at CpG sites (5mC) is established by DNMT3A/3B, maintained by DNMT1, and removed via TET1/2/3-mediated oxidation (5hmC, 5fC, 5caC). ncRNAs include lncRNA, miRNA, and circRNA, which regulate transcription and post-transcriptional processes. Together, these layers coordinate gene expression. Abbreviations: TAD, Topologically associating domain; CTCF, CCCTC-binding factor; TFs, Transcription factors; DNMT, DNA methyltransferase; DNMT1, DNA methyltransferase 1; DNMT3A/3B, DNA methyltransferase 3A/3B; TET, Ten-eleven translocation methylcytosine dioxygenase; 5mC, 5-methylcytosine; 5hmC, 5-hydroxymethylcytosine; 5fC, 5-formylcytosine; 5caC, 5-carboxylcytosine; H3K4me3,Trimethylation of histone H3 lysine 4; H3K27me3, Trimethylation of histone H3 lysine 27; Ac, Histone acetylation; Ub, Ubiquitination; ncRNA, Non-coding RNA; lncRNA, Long non-coding RNA; miRNA, MicroRNA; circRNA, Circular RNA.

Epigenetic regulation encompasses multiple mechanisms, each with distinct characteristics in terms of regulatory scope, persistence, and tissue specificity. This section provides an overview of the main features and regulatory roles of DNA methylation, histone modifications, non-coding RNAs, and chromatin structure-related mechanisms (Figure 2 and Supplementary Figure S1).

### 2.1. DNA Methylation

DNA methylation is one of the most typical and well-studied forms of epigenetic modification, primarily existing as 5-methylcytosine (5mC) in the genomes of eukaryotes. This modification is catalyzed by DNA methyltransferases (DNMTs), with S-adenosylmethionine (SAM) serving as the universal methyl donor for most cellular methylation reactions. With DNA methylation dynamics involving both maintenance methylation, primarily mediated by DNMT1 during DNA replication, and de novo methylation established by DNMT3A and DNMT3B with regulatory support from DNMT3L, this regulatory system exhibits tightly controlled enzymatic coordination [19]. DNA demethylation occurs through passive and active mechanisms. Passive demethylation results from the dilution of methylation marks during cell division when DNMT1 activity is reduced, whereas active demethylation is mainly mediated by the ten-eleven translocation (TET1/2/3) enzymes, which iteratively oxidize 5mC to 5-hydroxymethylcytosine (5hmC), 5-formylcytosine (5fC), and 5-carboxylcytosine (5caC), followed by thymine DNA glycosylase (TDG)-dependent base excision repair to restore unmethylated cytosines [20]. Additional evidence suggests that other DNA damage-repair pathways may also contribute to active DNA demethylation [21].

In mammals, DNA methylation is predominantly found at CpG dinucleotide sites [22,23]. In ruminants such as cattle, CpG islands (CGIs) account for only ~1% of the genome, yet they represent major regulatory hotspots. Genome-wide profiling studies show that CpG sites are enriched within CpG islands, CGI shores, and promoter regions, consistent with their regulatory roles in gene expression [9]. In addition to the classical CpG methylation, non-CpG methylation (mCH) is also prevalent in specific cell types such as pluripotent cells, the nervous system, and germ cells, exhibiting distinct tissue- and developmental-stage-specific patterns [24].

At the genomic regulatory level, DNA methylation plays differential roles in various functional regions. Methylation of CpG islands in the promoter region is closely associated with gene transcription, with unmethylated states typically linked to transcriptional activation, while hypermethylation is associated with transcriptional repression and chromatin compaction [25,26,27]. DNA methylation within enhancers and gene bodies exerts locus-specific regulatory effects on transcription beyond classical promoter repression. Longitudinal endothelial methylome profiling demonstrated dynamic enhancer hyper- and hypomethylation during angiogenic-to-quiescent transitions, which correlated with gene expression changes and chromatin accessibility. In parallel, sequential ChIP-bisulfite sequencing in differentiating mouse embryonic stem cells showed preferential CpG methylation gain at H3K4me1-marked enhancers relative to H3K27ac-marked active enhancers, indicating enhancer-state-dependent methylation heterogeneity linked to transcriptional variability. These studies provide mechanistic evidence that regulatory-element-specific methylation remodeling contributes to differentiation-associated transcriptome reprogramming [28,29]. Whole-genome bisulfite sequencing during yak adipocyte differentiation revealed that CG methylation levels within promoter regions were negatively correlated with gene expression. Integrative analysis with chromatin accessibility data further showed that differentially methylated regions overlapped with ATAC-seq peaks, where higher accessibility corresponded to lower methylation levels. These findings suggest coordinated, locus-specific associations between DNA methylation and chromatin accessibility during adipogenic gene regulation [30].

Numerous studies show that DNA methylation is highly sensitive to nutritional status, environmental stress, and developmental stages, often working synergistically with other epigenetic marks to regulate key biological processes in ruminants, such as spermatogenesis, embryonic development, muscle growth, and lipid metabolism [31,32]. At the mechanistic level, these dynamic methylation–demethylation processes frequently converge on key metabolic and developmental regulators, suggesting coordinated pathway-level regulation rather than isolated gene effects [33,34]. Therefore, DNA methylation is not only a crucial mechanism linking environmental factors to gene expression regulation but also one of the most mature and promising research directions in the current field of epigenetics in ruminants.

### 2.2. Histone Modifications

In eukaryotes, DNA exists in the form of chromatin, with its basic structural unit being the nucleosome, composed of a histone octamer (two copies of each H2A, H2B, H3, and H4) and the DNA wrapped around it [35]. Histones, especially their N-terminal tails, undergo various post-translational modifications (PTMs), such as methylation, acetylation, phosphorylation, ubiquitination, and a range of novel acylation modifications (2-hydroxyisobutyrylation, succinylation and lactylation) [36]. These modifications play a critical role in gene expression regulation by altering chromatin conformation or controlling the recruitment of transcription factors and regulatory complexes [37,38,39]. Histone modifications are regulated by coordinated ‘writer’ enzymes (e.g., HATs for acetylation, HMTs for methylation) that add marks, ‘eraser’ enzymes (e.g., HDACs for deacetylation, demethylases for demethylation) that remove them, and ‘reader’ proteins that recognize specific marks and recruit downstream effectors.

Histone methylation primarily occurs on lysine and arginine residues, with its biological function depending on the specific modification sites and the degree of methylation. Different marks can correspond to transcriptional activation or repression states [40]. Histone acetylation is typically catalyzed by histone acetyltransferases (HATs), which neutralize the positive charge on histones, enhance chromatin openness, and promote transcription. Conversely, histone deacetylases (HDACs) mediate the removal of acetyl groups, often associated with transcriptional repression [41]. Typical activation marks, such as H3K4me3 and H3K27ac, are enriched in promoter and active enhancer regions and are closely linked to transcription initiation and enhancer activity [42]. In addition to methylation and acetylation, other histone modifications, including phosphorylation, ubiquitination, and ADP-ribosylation, are involved in key biological processes such as DNA damage response, chromatin remodeling, and transcriptional regulation [43,44,45]. Different histone modifications often co-exist or antagonize each other within the same chromatin region, collectively regulating chromatin states through mutual interactions. For example, certain ubiquitination marks facilitate the establishment of subsequent methylation marks, while dynamic changes between methylation and acetylation modifications regulate the active states of promoter and enhancer regions [46,47].

In ruminants, histone modifications are highly sensitive to nutritional status, developmental stages, and environmental stress, and they work synergistically with DNA methylation and non-coding RNA regulation to participate in processes such as embryonic development, tissue differentiation, metabolic regulation, and environmental adaptation [48]. Therefore, histone modifications, as a highly dynamic and adaptable epigenetic mechanism, provide an important molecular foundation for understanding the formation of complex traits and environmental responses in ruminants.

### 2.3. Non-Coding RNAs

Non-coding RNA (ncRNA) is a class of functional RNA molecules transcribed from the genome but not translated into proteins. ncRNAs are widely involved in gene expression regulation and play crucial roles in biological processes such as growth and development, reproductive control, immune response, and environmental adaptation [49,50]. As an essential component of the epigenetic regulatory system, ncRNAs mediate multi-layered regulation at the transcriptional, post-transcriptional, and chromatin levels, enhancing the precision and plasticity of gene expression regulation [51,52].

Based on molecular length and functional characteristics, ncRNAs can be broadly categorized into long non-coding RNAs (lncRNAs) and small non-coding RNAs. Small regulatory ncRNAs include microRNAs (miRNAs), small interfering RNAs (siRNAs), and PIWI-interacting RNAs (piRNAs), which primarily participate in post-transcriptional gene silencing and transposon regulation. In contrast, small nucleolar RNAs (snoRNAs) are structurally and functionally distinct, mainly guiding rRNA and snRNA modification and contributing to ribosome biogenesis. Circular RNAs (circRNAs) represent another class of ncRNAs with regulatory roles in gene expression [53,54,55]. Different types of ncRNAs exhibit distinct mechanisms of action, yet they often collaborate in the same regulatory processes. miRNAs typically mediate post-transcriptional gene silencing by binding complementarily to target mRNAs, playing key roles in cell proliferation, differentiation, and metabolic regulation [56,57]. lncRNAs have more diverse mechanisms of action, including recruiting chromatin modification complexes, acting as competitive endogenous RNAs (ceRNAs) to regulate miRNA activity, or participating in chromatin spatial structure regulation, thus influencing gene transcription programs [58,59]. In contrast, circRNAs, due to their stable circular structure, often act as miRNA sponges or protein interaction platforms involved in gene expression regulation and function as functional peptides in specific physiological and developmental contexts [60,61].

Mechanistically, a subset of ncRNAs can act as regulatory bridges coordinating DNA methylation, histone modification, and chromatin architecture through the recruitment of epigenetic complexes to specific genomic loci, thereby contributing to the integration of environmental signals into transcriptional programs. In ruminants, ncRNAs are closely associated with growth and development, reproductive performance, immune homeostasis, and livestock product traits, exhibiting distinct dynamic regulatory characteristics under nutritional changes and environmental stress. Therefore, ncRNAs, as highly adaptable epigenetic regulators, provide important insights for understanding the mechanisms behind the formation of complex traits in ruminants and for exploring new breeding regulation targets.

### 2.4. Chromatin Remodeling and 3D Genome Architecture

In eukaryotes, chromatin remodeling influences transcription by regulating nucleosome positioning and chromatin accessibility, thereby affecting the binding of transcription factors and regulatory complexes to gene regulatory sequences, thus participating in the dynamic regulation of gene expression [62]. Building on chromatin accessibility regulation, the eukaryotic genome also exhibits a highly organized three-dimensional spatial structure within the nucleus. Linear DNA folds hierarchically to form multi-scale spatial structural units, including chromosome territories (CTs), chromatin compartments (A/B compartments), topologically associating domains (TADs), and chromatin loops [63]. These spatial structures either restrict or promote interactions between enhancers and promoters, providing a spatial basis for the regulation of gene expression [64]. Chromatin remodeling and 3D genome architecture are not independent; rather, they interact with epigenetic marks such as DNA methylation and histone modifications, collectively shaping chromatin conformation and the gene regulatory environment [65,66].

At the molecular level, chromatin remodeling is mediated by ATP-dependent remodeling complexes such as SWI/SNF, ISWI, and CHD families, which reposition or evict nucleosomes to regulate DNA accessibility [67]. In 3D genome organization, architectural proteins such as CTCF and cohesin play essential roles in establishing chromatin loops and TAD boundaries, thereby stabilizing enhancer–promoter interactions and coordinating long-range gene regulation [68]. In ruminants, chromatin accessibility and 3D genome architecture are closely associated with embryonic development, tissue differentiation, metabolic regulation, and environmental adaptation, exhibiting dynamic responses to nutritional changes and environmental stress [69,70]. Therefore, chromatin remodeling and 3D genome architecture, as integral components of epigenetic regulation, provide key spatial regulatory insights for understanding the formation of complex traits in ruminants and their mechanisms of environmental response.

## 3. Advances in Epigenetic Studies in Ruminants

The growth and development, reproductive performance, metabolic and immune homeostasis, and livestock product traits of ruminants represent key trait types that span from fundamental biological processes to production outcomes. These traits exhibit significant variations across different tissues, developmental stages, and environmental conditions. Their formation relies not only on genetic background but also on the combined influence of multiple regulatory factors. Increasing evidence suggests that epigenetic marks exhibit characteristic changes in relevant tissues and physiological stages, and contribute to the formation of trait differences by regulating gene expression. This section synthesizes research evidence on the relationship between epigenetic marks and trait expression for the aforementioned core traits, with a focus on analyzing the roles and regulatory patterns of different epigenetic mechanisms in various traits, providing evidence for the subsequent application of epigenetic information in breeding decisions and production management.

### 3.1. The Impact of Epigenetic Marks on Growth and Development in Ruminants

Growth and development are fundamental processes underlying the formation of production traits in ruminants, with muscle growth and fat deposition directly determining meat production potential and meat quality characteristics. In addition to genetic variation, an increasing body of research shows that epigenetic marks undergo dynamic changes across different developmental stages and tissue types. These marks regulate gene networks associated with cell proliferation, differentiation, and metabolism, thereby contributing to the regulation of growth and development.

#### 3.1.1. Epigenetic Mechanisms Underlying Skeletal Muscle Growth and Development

During muscle development, the proliferation, differentiation, and hypertrophy of muscle fibers are continuous and interconnected key biological events, regulated by stage-specific gene expression programs. During sheep skeletal muscle development, whole-genome bisulfite sequencing identified seven *GTL2*-associated DMRs exhibiting higher methylation levels in adult compared with fetal longissimus dorsi muscle, with methylation increases ranging from 31.1% to 61.8% and the largest change observed at DMR7 (+61.8%). Targeted epigenome editing elevated DMR7 methylation by 8.9% and 37.5% and was accompanied by reduced *GTL2* expression, whereas demethylation treatment increased transcript abundance in a dose-dependent manner, indicating that increased methylation of the intronic DMR7 region influences *GTL2* transcription during skeletal muscle development [71]. Further chromatin-level studies revealed that muscle cell differentiation is accompanied by significant chromatin accessibility remodeling. Combined ATAC-seq and RNA-seq analysis of skeletal muscle satellite cells from Altai sheep identified 17,460 differential accessible regions, with 11,037 regions remaining open during differentiation. Key regulatory pathways, including PI3K–Akt, TGF-β, calcium signaling, and ECM–receptor interaction, form a core regulatory network that drives the transition from proliferation to differentiation. Additionally, key candidate genes, such as *FZD5* and *MAP2K6*, were identified, providing potential targets for molecular breeding of sheep muscle traits [72].

#### 3.1.2. Epigenetic Mechanisms Underlying Adipogenesis and Fat Deposition

Fat deposition, a highly tissue-specific and phenotypically variable trait during growth and development, is also regulated by epigenetic mechanisms. Whole-genome DNA methylation analysis using MeDIP-seq on sheep with different tail types (fat-tailed, semi-fat-tailed, and thin-tailed) revealed a close association between tail type and DNA methylation patterns. Differentially methylated regions (DMRs) were mainly enriched in intronic and exonic regions. These DMRs are associated with fat metabolism-related genes, including *NFATC4*, *LPIN2*, *MGAT2*, and *MAT2B*, suggesting a potential link between DNA methylation variation and tail fat deposition [73]. During the differentiation of yak adipocytes, dynamic changes in chromatin accessibility were highly coordinated with the expression of lipid metabolism genes. The transcription factors FOS and JUNB significantly affected the differentiation and lipid deposition of plateau yaks by regulating the open state of promoter regions [74]. Furthermore, in the study of intramuscular fat deposition in sheep, seven candidate lncRNAs were identified, which may regulate the expression of target genes through lncRNA-mRNA co-expression networks, such as MSTRG.4051.3-FZD4, thereby participating in the regulation of intramuscular lipid deposition [75].

Studies in sheep, cattle, and yak indicate that epigenetic regulation is closely involved in muscle development and adipogenesis, with epigenetic marks showing clear developmental-stage specificity, particularly during the transition from proliferation to differentiation in muscle and adipose tissues (Table 1 and Supplementary Table S1). However, most evidence is based on tissue- or stage-specific profiles, making it difficult to distinguish causal regulatory roles from epigenetic changes secondary to transcriptional remodeling, and functional validation across genetic backgrounds remains limited. Epigenetic marks like DNA methylation and histone modifications are linked to muscle growth and fat development, but these associations are mostly correlational. Their stability and heritability, especially under different environmental conditions, need further investigation.

**Table 1:** Epigenetic regulation of muscle development and adipogenesis in ruminants.

Traits	Tissue/Cell Type	Key Technologies	Epigenetic Marks	Key Gene/Pathways	References
Muscle growth and development	Longissimus dorsi muscle, Skeletal muscle satellite cells	ATAC-seq, RNA-seq, Small RNA-seq, circRNA seq, WGBS	Chromatin accessibility and miRNA-mediated regulation, lncRNA-mediated transcriptional regulation (cis/trans), circRNA-mediated miRNA sponging, DNA methylation and promoter chromatin accessibility dynamics	*SIX1*, *NEDD4L*, *SCN3B*, *HDAC4*, *ACACB*, *TGFβ2*, *MyoD*, *MYOG*, *FADS1*, *SLC25A1*; Pathways: Hippo.Ras, MAPK, Wnt, FoxO AMPK, PI3K-Akt, PPAR, Hippo, TGF-beta	[76,77,78]
Adipogenesis and fat deposition	Biceps femoris muscle, Longissimus thoracis muscle, adipose tissue	RNA-Seq, WGBS	lncRNA-miRNA-mRNA (ceRNA) regulatory network, Gene body DNA methylation associated with lipid metabolism	*HADHA*, *CPT1A*; *ACSM1*, *EHHADH*, *BDH1*, *APLNR*, *GPLD1*, *S100A9*; PPAR signaling pathway	[79,80]

Note: Summary of representative epigenetic studies on muscle development and adipogenesis in ruminants. The table summarizes the species, tissue or cell type, epigenetic layer, analytical methods, and key biological findings related to growth- and fat deposition-associated traits. Species abbreviations and classifications apply consistently across Table 1, Table 2, Table 3 and Table 4. Abbreviations: ATAC-seq, Assay for transposase-accessible chromatin using sequencing; RNA-seq, RNA sequencing; Small RNA-seq, Small RNA sequencing; circRNA-seq, Circular RNA sequencing; WGBS, Whole-genome bisulfite sequencing.

### 3.2. The Impact of Epigenetic Marks on Reproductive Performance in Ruminants

Reproductive performance is a key trait in ruminant production systems, and its formation involves a series of highly intricate and stage-specific biological events, including gametogenesis, fertilization, embryonic development, and implantation. In addition to genetic factors, an increasing body of research suggests that epigenetic marks exhibit dynamic changes across different sexes, reproductive tissues, and developmental stages. These marks regulate key genes and signaling pathways, thereby contributing to the regulation and differentiation of reproductive traits.

#### 3.2.1. Epigenetic Mechanisms Underlying Male Reproductive Traits

In male ruminants, testicular development, spermatogenesis, and semen quality are critical factors determining reproductive performance. Studies show that DNA methylation participates in the regulation of sperm function by affecting gene structure and transcriptional regulation. For example, DNA methylation at exon 29 of the *PBRM1* gene is associated with selective splicing events and differences in sperm structure and motility, suggesting a potential role in semen quality variation in cattle [81]. At the post-transcriptional level, transcriptome profiling and lncRNA–mRNA interaction network analyses in dairy goat seminiferous tubules identified 229 spermatogenesis-related genes and inferred candidate ncRNA–gene associations involving *Piwil1*, *Piwil2*, and *Gtsf1* that were linked to seminiferous tubule maturation [82]. In parallel, small RNA sequencing of ram sperm revealed 227 differentially expressed miRNAs, including oar-miR-200b and oar-miR-26b, with predicted target genes enriched in pathways related to ribonucleoprotein complex biogenesis and RNA processing. Together, these findings indicate association-based relationships between sperm sncRNA expression profiles and sperm functional characteristics, supporting their potential utility as molecular biomarkers of male fertility [83]. Comparative studies across mammals indicate that core epigenetic regulatory frameworks of sperm development, including post-transcriptional regulation mediated by small RNAs, are broadly conserved between ruminants and model organisms such as mice and humans. However, cross-species sperm methylome analyses have revealed that specific regulatory regions and epigenetic marks are largely species-specific, reflecting divergent reproductive physiology and evolutionary pressures, as demonstrated by distinct sperm DNA methylation patterns among human, bovine, and mouse germlines [84].

#### 3.2.2. Epigenetic Mechanisms Underlying Female Reproduction and Sex Control

In female ruminants, ovarian follicle development, fertilization, early embryonic development, and implantation collectively determine reproductive efficiency. Studies show that epigenetic regulation plays a crucial role in the follicular microenvironment and embryonic development. In goats, seven miRNAs, including miR-202-5p, are significantly upregulated in extracellular vesicles (EVs) derived from large follicles and promote follicle development by regulating the PI3K-AKT signaling pathway [85]. In bovine follicular fluid, miR-29b, miR-199a-3p, and miR-148a can enhance early embryo DNA demethylation by inhibiting the expression of DNA methyltransferases (DNMTs), thereby improving embryo developmental quality [86]. At the epigenetic reprogramming level, inhibition of LSD1 using 2-PCPA increased H3K4me2 levels by approximately 1.4–1.6-fold in donor fibroblasts, accompanied by enhanced expression of pluripotency genes (*Oct4* and *Sox2*) and improved in vitro developmental rates of goat SCNT embryos [87].

In addition to conventional reproductive processes, sex control, as an important technical tool in ruminant reproductive management, holds special significance in dairy production. Studies show that there are 12,175 differentially methylated regions (DMRs) between the X and Y sperm of Holstein bulls. These regions are predominantly enriched in genes related to energy metabolism and sperm function, such as *SPA17*, *PLCB1*, and *PEG3*, revealing systemic epigenetic differences between X and Y sperm. These findings provide molecular evidence for the development of new sex control strategies [88].

Available evidence indicates that epigenetic regulation is closely involved in ruminant reproductive processes, particularly during gametogenesis and early embryonic development. Across studies, DNA methylation and small RNA-mediated regulation are repeatedly associated with sperm quality, follicular development, and embryo competence (Table 2 and Supplementary Table S2). However, the stability and heritability of these epigenetic marks remain uncertain. Many reported associations are strongly dependent on reproductive stage, cell type, or assisted reproductive procedures, and causal links to reproductive outcomes are still largely inferred rather than experimentally validated.

**Table 2:** Epigenetic regulation of reproductive traits in ruminants.

Traits	Tissue/Cell Type	Key Technologies	Epigenetic Marks	Key Gene/Pathways	References
Spermatogenesis and male fertility	Seminiferous tubules, Germ cells, Sperm	RNA-seq, scRNA-seq, EM-seq	lncRNA-mediated transcriptional regulation, DNA methylation associated with sex chromosome-linked fertility traits	*Piwil1/2/4*, *Dnmt3l*, *Gtsf1*, *Ddx4*, *Sycp1*, *OR6A2*, *OR3A1*, *SPATA1*, *ADCY9*, *PDGFRA*; Pathways: PI3K/Akt, MAPK, Rap1, development-related pathways	[82,89]
Follicular development and female fertility	Follicular fluid extracellular vesicles	scRNA-seq	miRNA-mediated regulation of follicular development	Pathways: FoxO, MAPK, PI3K-AKT	[85]
Early embryonic development	Oocytes and early embryos	WGEMS, ddPCR	Mitochondrial DNA methylation during early embryogenesis	Pathways: ND6, CYTB	[90]
Reproductive endocrine regulation and sexual maturation	Hypothalamus, distal pituitary	RNA-seq, miRNA-seq	lncRNA-mediated transcriptional regulation (cis/trans), miRNA-mediated regulation of estrus and reproductive endocrine function	*LHB*, *TSHB*, *OXT*, *GH*, *Lhx1*, *DENND1A*, *EML6*, *SIX1*; Pathways: GnRH, Wnt, ErbB, circadian rhythm pathway, GnRH signaling, p53 signaling	[91,92]

Note: Summary of representative epigenetic studies on ruminant reproduction. The table summarizes the species, reproductive tissue or cell type, epigenetic mechanisms, experimental approaches, and associated reproductive traits, including gametogenesis, follicular development, embryo competence, and sex-related differences. Abbreviations: RNA-seq, RNA sequencing; scRNA-seq, Single-cell RNA sequencing; EM-seq, Enzymatic methyl sequencing; WGEMS, Whole-genome enzymatic methyl sequencing; ddPCR, Droplet digital PCR; miRNA-seq, MicroRNA sequencing.

### 3.3. The Impact of Epigenetic Markers on Metabolism, Immunity, and Diseases in Ruminants

Metabolic homeostasis and immune function are crucial physiological foundations for maintaining the health and production performance of ruminants. These two processes are highly interconnected at the molecular regulatory level and jointly influence the individual’s susceptibility to diseases. Recent studies show that epigenetic markers, by regulating the expression of metabolism-related genes and immune response pathways, play a role in metabolic regulation, immune homeostasis maintenance, and disease progression across different tissues and physiological states.

#### 3.3.1. Epigenetic Mechanisms Underlying Metabolic Homeostasis in Ruminants

In metabolic regulation, histone modifications are implicated in milk fat metabolism and energy balance. In goat mammary epithelial cells, epigenetic activation signals, including H3K27ac enrichment at the *FoxO1* locus, are associated with *FoxO1* binding to the FKH2 site in the *ATGL* promoter and increased *ATGL* transcription, suggesting a potential role in milk fat hydrolysis. Meanwhile, insulin signaling through the PI3K-AKT pathway induces *FoxO1* phosphorylation and nuclear–cytoplasmic translocation, which may attenuate *FoxO1*-dependent transcriptional activity and contribute to the maintenance of milk fat metabolic homeostasis [93]. This study highlights the synergistic regulation of milk fat metabolism in ruminants by histone modifications and classical metabolic signaling pathways.

#### 3.3.2. Epigenetic Mechanisms Linking Immunity and Disease Susceptibility

In immune regulation, changes in chromatin state and spatial structure have a significant impact on the expression of immune-related genes. In sheep alveolar macrophages, active histone modifications such as H3K4me3 and H3K27ac mark promoter and enhancer regions, and work in synergy with CTCF-mediated chromatin three-dimensional domains to maintain the transcriptional activity of immune-related genes. In contrast, H3K27me3 predominantly accumulates in the regions of development-related genes and mediates their transcriptional silencing, revealing the differential regulatory roles of various epigenetic marks on gene expression in immune cells [94].

Abnormalities in metabolic and immune regulation are often closely associated with disease susceptibility. In ruminant mastitis, changes in DNA methylation are significantly correlated with immune response and production performance. Studies have reported that in cows with *S. aureus* subclinical mastitis, genome-wide methylation profiling identified 153,783 differential methylation haplotype blocks (dMHBs), including subsets located within regulatory regions of metabolism- and immune-related genes such as *CPT1A* and *TRAK1*. These methylation alterations showed measurable inverse associations with milk yield and mammary health traits, although current evidence remains primarily correlational and does not establish whether these methylation differences represent causal regulatory drivers of disease susceptibility or secondary consequences of inflammatory processes [95]. Furthermore, *Staphylococcus aureus* cell wall components PGN and LTA regulate DNA methylation and histone H3 acetylation levels, influencing the expression of inflammatory cytokines such as IL-1β and IL-6, as well as casein genes *CSN2* and *CSN3*, thereby inducing mastitis and reducing lactation function [96]. In the cecal valve tissues of cows infected with *Mycobacterium avium* subsp. *paratuberculosis* (MAP), multiple differentially expressed lncRNAs were identified within QTL regions associated with various diseases such as mastitis and tuberculosis. These lncRNAs were significantly correlated with immune-regulatory genes such as *MTMR9*, *RGMB*, and *HOXA6*, suggesting that non-coding RNAs may play a role in the epigenetic regulation of disease susceptibility in ruminants [97].

Evidence across studies indicates that epigenetic modifications are closely associated with metabolic and immune states in ruminants, particularly under nutritional challenge or disease stress, supporting a role for epigenetic regulation in metabolic and immune plasticity (Table 3 and Supplementary Table S3). Epigenetic modifications influence metabolism and immunity, but many associations are tissue- and environment-dependent. Further studies are needed to confirm causal relationships. However, most findings remain correlational, and it is often unclear whether observed changes in DNA methylation or histone modifications represent primary regulatory events or downstream consequences of inflammation and metabolic disturbance. Longitudinal and intervention-based studies are therefore needed to clarify causal relationships and the relevance of epigenetic markers for disease susceptibility and resilience.

**Table 3:** Epigenetic regulation of metabolism, immunity, and disease in ruminants.

Traits	Tissue/Cell Type	Key Technologies	Epigenetic Marks	Key Gene/Pathways	References
Milk fat metabolism Immune response	Goat mammary epithelial cells, Alveolar macrophages, Lymph nodes, abomasum, duodenum	ChIP-seq, RNA-seq, Mammalian methylation array	H3K9ac H3K4me3, H3K27ac, H3K4me1, H3K27me3, DNA methylation	*FASN*, *SCD1*, *FADS1*, *LPIN1*, *DGAT1*, *MBOAT2,*	[94,98,99]
				*PPARG*, *BHLHE40/41*, *SATB1*, *LRFN5*; Pathways: Wnt/β-catenin, Th1/Th2, RAR, NGF, IL	
Disease susceptibility and pathogenesis	Peripheral blood, Brain (Thalamus), Ileocecal valve	WGBS, IHC, RT-qPCR, RNA-Seq	DNA methylation, Decreased 5mC & 5hmC levels, lncRNA-mediated transcriptional regulation	*IL1R1*, *BOLA-DQB*, *DNMT3B*, *HDAC2*, *TET1*, *MTMR9*, *RGMB*, *IL-6*, *PRNCR1*, *HOXA-AS3*; Pathways: Calcium signaling, MAPK signaling, Metabolic pathways	[97,100,101]

Note: Summary of representative epigenetic studies on metabolic regulation, immune responses, and disease susceptibility in ruminants. The table summarizes the species, tissue or cell type, epigenetic layer, analytical platforms, and major associations with metabolic and immune traits, including mastitis and infectious diseases. Abbreviations: ChIP-seq, Chromatin immunoprecipitation sequencing; RNA-seq, RNA sequencing; WGBS, Whole-genome bisulfite sequencing; IHC, Immunohistochemistry; RT-qPCR, Reverse transcription quantitative PCR.

### 3.4. The Impact of Epigenetic Marks on Ruminant Livestock Product Traits

Livestock product yield and quality are the most direct economic traits in ruminant production systems and serve as the core objectives in genetic improvement and breeding practices. The formation of livestock product traits such as dairy products, meat, and wool involves various biological processes, including mammary gland function, muscle and fat development, and hair follicle biology. Recent studies show that epigenetic regulation in these processes has gained increasing attention and is considered one of the key molecular foundations influencing the variation in livestock product traits.

#### 3.4.1. Epigenetic Mechanisms Underlying Dairy Production Traits

In dairy traits, DNA methylation and non-coding RNAs are considered important regulatory factors affecting milk yield and milk composition. In Zaraibi goats, CpG island methylation within the promoter regions of the milk production-related genes *GHR* and *GDF9* differed between high- and low-yield animals. Promoter methylation, measured using methylation-specific PCR and combined bisulfite restriction analysis, was positively correlated with gene expression and milk yield traits. However, concurrent effects of breeding season and parity indicate that these methylation patterns represent association-based epigenetic markers rather than direct causal determinants of milk production [102]. In Holstein cows, whole-genome bisulfite sequencing identified differentially methylated CpG sites associated with milk fat and protein traits, with several methylation signals co-located with quantitative trait loci linked to milk component yield. These findings indicate potential epigenetic associations between regional DNA methylation patterns and variation in milk composition [103]. Additionally, functional studies in bovine mammary epithelial cells demonstrated that miR-34b modulates milk fat synthesis through a validated miR-34b-RAI14-Akt/mTOR regulatory axis, indicating that specific miRNAs participate in the regulation of lipid metabolism-related gene expression in mammary tissue [104].

#### 3.4.2. Epigenetic Mechanisms Underlying Meat Quality and Carcass Traits

In meat traits, DNA methylation and ncRNA regulation play important roles in muscle development and fat deposition processes. Studies show that multiple lncRNAs regulate intramuscular fat deposition by modulating fat-related transcription factors, such as C/EBPα and PPARγ. For instance, BIANCR affects adipogenesis through the ERK1/2 signaling pathway [105,106]. Additionally, differential DNA methylation in key mitochondrial β-oxidation genes, including *ACSM1*, *EHHADH*, and *BDH1*, is associated with reduced gene expression and decreased lipid oxidation capacity, which may contribute to intramuscular fat accumulation and meat quality variation in grass-fed cattle during the finishing phase [80]. Moreover, RNA-seq analysis reveals that several differentially expressed lncRNAs, such as lncRNA_15786.3, regulate genes involved in lipid metabolism, including *CCN1*, *BNIP3*, and *CNOT2*, influencing the composition of polyunsaturated fatty acids (PUFAs) and saturated fatty acids (SFAs) in bovine muscles [107]. In terms of fine meat quality traits, integrating Iso-seq, RNA-seq, and CTCF ChIP-seq studies has revealed that CTCF may influence the selective splicing of *ANKRD23* by regulating DNA methylation status, thus contributing to lamb meat tenderness [108].

#### 3.4.3. Epigenetic Mechanisms Underlying Wool and Fiber Quality Traits

In addition to dairy and meat products, pelts (wool) are also significant economic products of ruminants. Studies show that hair follicle development, fiber growth, and the quality traits of hair fibers (such as fineness and curvature) are influenced by DNA methylation, histone modifications, and ncRNA regulation [109,110]. Genome-wide methylation and transcriptomic analyses have identified multiple genes involved in hair follicle development, including *WNT2*, *EDN1*, *LAMC2*, *NR1D1*, *RORB*, and *MYOZ3*, showing coordinated differential methylation and expression across developmental stages. These genes are enriched in signaling pathways such as Wnt, TNF, TGF-β, MAPK, and ECM-receptor interaction, with methylation-mediated activation of *WNT2* promoting fibroblast proliferation and supporting hair follicle development [111]. In addition, studies indicate that the imprinted *Gtl2*-miRNA locus plays a critical role in regulating primary hair follicle formation and wool type differentiation. miRNAs derived from this locus suppress the PI3K/AKT/mTOR signaling pathway, thereby reducing oxidative stress and apoptosis in hair follicle stem cells while promoting follicle proliferation and regeneration. Furthermore, a newly identified sITS-derived miRNA cluster acts synergistically with *Gtl2*-miRNAs to modulate stem cell activity and hair follicle morphogenesis [112]. These epigenetic changes play an important role during hair follicle morphogenesis and fiber type differentiation.

Evidence across dairy, meat, and fiber traits indicates that epigenetic regulation contributes to variation in product yield and quality through tissue-specific gene expression programs. Epigenetic marks are linked to product traits such as milk yield and meat quality, but these associations are often descriptive. Their stability and heritability in commercial breeding require more validation. Across studies, DNA methylation and ncRNA-mediated regulation frequently converge on pathways related to lipid metabolism, cell differentiation, and extracellular matrix organization (Table 4 and Supplementary Table S4). At the same time, epigenetic regulatory patterns are highly trait- and tissue-specific, which limits the transferability of individual epigenetic markers across production traits and highlights the need for trait-focused functional validation before their use in breeding programs.

Collectively, these findings suggest that epigenetic mechanisms modulate coordinated gene networks rather than single genes, thereby shaping complex traits such as growth, reproduction, and immune function. The functional implications of these epigenetic mechanisms for genetic evaluation and breeding applications are further discussed in Section 4.

**Table 4:** Epigenetic regulation of ruminant livestock product traits.

Traits	Tissue/Cell Type	Key Technologies	Epigenetic Marks	Key Gene/Pathways	References
Milk production and composition	Mammary tissue, Bovine mammary epithelial cells	MSP, COBRA, RNA-seq	DNA methylation-mediated regulation, miRNA-mediated post-transcriptional regulation	*GDF-9*, *GHR*, *RAI14*; Akt/mTOR signaling pathway	[102,104]
Meat quality traits	Heart, liver, spleen, lung, kidney, muscle, adipose, Longissimus dorsi muscle, Longissimus lumborum/thoracis muscle	RNA-seq, WGCNA, MethylRAD, MBD-seq, RRBS	ncRNA-mediated regulation of lipid deposition, DNA methylation associated with muscle development, DNA methylation associated with meat tenderness	*PLIN1*, *CDK6*, *G3BP1*, *MAPK4*; *IGF2*, *TMEM8C*, *CACNA1S*, *ABCG1*, *MYH8*, *MYO5A*; Pathways: PPAR, insulin, AMPK, MAPK, PI3K-Akt signaling, Metabolic pathways, G protein signaling, cAMP	[113,114,115,116]
Hair follicle development and fiber traits	Skin tissue, hair follicle, dermal papilla cells	RNA-seq, IHC, RT-qPCR	lncRNA-mediated regulation of hair follicle development	*KRTAP15-1*, *FGF1*, *IGF1*, *RAC2*, *FOXN1*, *KRT71*, *KRT82*, *LIPK*, *DNASE1L2*; Pathways: Wnt, PI3K-Akt, Ras, MAPK	[117,118]

Note: Summary of representative epigenetic studies on ruminant livestock product traits. The table summarizes the species, target tissue, epigenetic regulatory layer, methodological approaches, and main associations with dairy and meat production traits, including milk yield and composition, intramuscular fat deposition, and meat quality. Abbreviations: MSP, methylation-specific PCR; COBRA, Combined bisulfite restriction analysis; RRBS, Reduced representation bisulfite sequencing; WGCNA, Weighted gene co-expression network analysis; MethylRAD, Methylation-dependent restriction site-associated DNA sequencing.

## 4. Application of Epigenetic Marks in Ruminant Breeding

As the regulatory role of epigenetics in various important traits of ruminants is gradually being elucidated, integrating these biological findings into breeding systems has become a core focus of genetic improvement research for ruminants. Building on the evidence of trait associations discussed earlier, this section explores the potential application of epigenetic information in breeding practices, examining its role in breeding decisions and strategy optimization, and analyzing its possible synergy with traditional genetic selection methods.

### 4.1. Epigenetic Biomarkers for Phenotype Prediction

Building on numerous studies that have clarified the regulatory role of epigenetic marks in key traits of ruminants (such as milk yield, growth rate, disease resistance, and reproductive performance), the application of this information for trait prediction and early screening is gradually becoming a significant focus in ruminant breeding. Epigenetic features such as DNA methylation, non-coding RNA, and histone modifications can reflect environmental stress and physiological state changes, providing an additional layer of information beyond DNA sequences for complex traits. This helps to overcome the limitations of traditional genetic selection in explaining phenotypic plasticity and environmental effects [119].

In recent years, several studies have validated the feasibility of epigenetic marks as predictive biomarkers. Based on sperm methylome analysis using reduced representation bisulfite sequencing (RRBS), 490 differentially methylated sites (DMCs) associated with fertility were identified in Montbéliarde bulls, and a predictive model was constructed using a random forest approach. The model achieved approximately 72% prediction accuracy in both the test set and independent validation set, indicating that sperm DNA methylation features can serve as effective indicators for assessing bull fertility [120]. Similarly, through blood DNA methylome analysis, researchers identified differential methylation sites and regions associated with mastitis resistance in dairy cows, and an early prediction of mastitis resistance was achieved using 50 core methylation markers [121]. Furthermore, sperm meQTL mapping studies showed that many methylation sites are regulated by genetic variation and are enriched in key regulatory regions, providing a new framework for the association analysis between epigenetic marks and genetic background [122]. Representative application cases are summarized in Table 5.

Despite the growing evidence supporting epigenetic biomarkers for phenotype prediction, several practical constraints currently limit their widespread application in ruminant breeding. First, many epigenetic marks exhibit strong tissue- and cell-type specificity, whereas breeding programs rely on easily accessible samples such as blood, sperm, milk somatic cells, or hair follicles. The extent to which epigenetic signals from these surrogate tissues accurately reflect regulatory states in target tissues remains uncertain and requires systematic validation. Second, epigenetic profiles are highly sensitive to environmental conditions, physiological status, age, and management practices, raising concerns about temporal stability and reproducibility across production systems. Third, the cost and logistical complexity of large-scale epigenomic profiling, together with the lack of standardized sampling and analytical pipelines, remain significant barriers to routine implementation. These constraints highlight the need to view epigenetic biomarkers as a complementary and context-dependent tool rather than a standalone solution in current breeding programs.

### 4.2. Integration of Epigenetic Information into Breeding Strategies

Traditional ruminant breeding mainly relies on genetic markers (e.g., SNPs) and genomic estimated breeding values (GEBVs) to improve genetic gain through genome-wide selection. However, such models often treat genotype–environment (G × E) interactions implicitly or with limited resolution and are therefore unable to fully capture dynamic environmental regulation of phenotypic expression [131,132]. In recent years, breeding models incorporating environmental factors, such as G × EBLUP, have significantly improved prediction accuracy by constructing environment-specific genomic relationship matrices, particularly when significant G × E interactions are present, highlighting the necessity of integrating environmental information into breeding decisions [133]. In contrast, epigenetic selection directly utilizes epigenetic markers to reveal environment-induced regulatory changes, providing a molecular basis for phenotypic plasticity. For example, DNA methylation-derived scores (EpiScores) represent weighted combinations of DNA methylation values across multiple CpG sites, analogous to polygenic scores in genomics. These scores have been evaluated as auxiliary predictors for fertility- and production-related traits, and their integration with genomic estimated breeding values has improved predictive performance in several livestock studies [134]. In addition, epigenetic markers associated with sperm quality and cryotolerance are increasingly considered in artificial insemination programs, representing early-stage translational applications of epigenetic information. Notably, the overlaps between genes and pathways identified by GWASs and EWASs are limited, indicating that genetic variation and epigenetic regulation capture different levels of biological information, making them distinctly complementary [132]. From a temporal perspective, traditional genetic selection relies on the long-term accumulation of genetic variation, while epigenetic regulation can respond to environmental changes within shorter generations. The combination of both approaches offers the potential to simultaneously address long-term genetic improvement and short-term environmental adaptation.

In addition, the application of epigenetic information in ruminant reproductive technologies has also garnered increasing attention. Studies show that DNA methylation stability during sperm cryopreservation is significantly correlated with post-thaw sperm quality and pregnancy rates. In Boer goats, optimizing the cryoprotectant formulation can effectively maintain sperm methylation levels and significantly improve pregnancy rates [135]. In somatic cell nuclear transfer (SCNT), the efficiency of epigenetic reprogramming is considered a key limiting factor for embryonic development. Research has found that specific long non-coding RNAs (such as lncRNA3720) can regulate histone variant expression and improve the developmental potential of cloned embryos [136].

From a practical perspective, the application of epigenetic information in ruminant breeding can be described as a stepwise process. Practically accessible samples (e.g., blood, sperm, or milk somatic cells) are collected under standardized conditions, followed by epigenetic profiling and basic quality control to ensure data comparability. Epigenetic features showing consistent trait associations are then evaluated for robustness and integrated with genomic and phenotypic information as a complementary, context-dependent layer for trait prediction.

Although epigenetic biomarkers show considerable promise for improving phenotype prediction, several barriers currently limit their large-scale application in ruminant breeding. These include high tissue specificity, sensitivity to environmental variation, and the lack of standardized sampling and analytical pipelines. Consequently, epigenetic information should currently be viewed as a complementary rather than a replacement layer to genomic selection, with its greatest value likely arising from integrative multi-omics breeding models.

## 5. Perspectives

Epigenetic regulation plays a significant role in mediating the effects of environmental factors on phenotypic variation in ruminants. Compared to the relative stability of DNA sequences, the epigenetic state is responsive to changes in external conditions such as nutritional intake, feeding management, and physiological stress, contributing to the development of phenotypic plasticity during individual development and production [137]. Current research continues to reveal that environmental factors, including specific nutritional components such as methyl donors (e.g., SAM, folate, methionine, choline, and vitamin B12), overall energy supply, and fatty acid composition, can influence growth, metabolism, and health in ruminants by regulating epigenetic mechanisms such as DNA methylation, histone modifications, and non-coding RNAs [138]. This dynamic regulatory feature provides important insights into the understanding of complex trait formation in ruminants.

With the continuous advancement of epigenetic marker research in ruminants, the identification of numerous potential regulatory targets has laid a theoretical foundation for the functional analysis and directional regulation of complex traits [139]. Epigenetic regulation thus serves as a conceptual complement to DNA sequence-based breeding approaches, expanding our understanding of how environmental exposures interact with regulatory networks to shape complex traits in ruminants [140]. Particularly in traits with low heritability and high environmental sensitivity, such as reproductive performance, metabolic efficiency, and stress adaptation, epigenetic information can more directly reflect an individual’s response to environmental changes [141]. Meanwhile, accumulating evidence indicates that environmental factors, such as nutritional regulation and stress exposure, influence trait formation through epigenetic mechanisms. This suggests that integrating epigenetic information into breeding decision-making systems could enhance the precision breeding and trait improvement pathways for ruminants [142,143]. Therefore, epigenetic information provides a critical functional-level supplement for the genetic evaluation and breeding decision-making of complex traits in ruminants.

Comparative epigenetic studies across mammals indicate that DNA methylation patterns exhibit both conserved and lineage-specific features. Cross-species analyses among ruminant livestock have shown that DNA methylation divergence is preferentially enriched at promoters and distal regulatory regions, where ruminant-specific hypomethylated regions are significantly associated with genes involved in metabolism, growth, and reproduction and are frequently colocalized with GWAS signals for economically important traits [144]. In parallel, comparative analyses of sperm DNA methylomes across cattle, mice, and humans demonstrate that, although global CpG methylation levels are broadly conserved, promoter methylation displays a bimodal distribution and pronounced species specificity: conserved hypomethylated promoters are enriched for genes essential for embryonic development, whereas cattle-specific hypomethylated promoters preferentially regulate lipid metabolism and production-related pathways [145]. Together, these findings indicate that conserved epigenetic mechanisms underlie core developmental processes, whereas species- and lineage-specific DNA methylation patterns contribute to phenotypic divergence and adaptive trait evolution in ruminants. Although this review synthesizes findings across ruminant species, epigenetic regulation may exhibit species-specific patterns influenced by differences in physiology, domestication history, tissue biology, and production systems. Accordingly, extrapolation across cattle, sheep, goats, and yaks should be interpreted with caution, and species-specific validation remains essential for translational breeding applications. Nevertheless, translating these insights into breeding practice will require coordinated methodological, economic, and regulatory advancements.

## 6. Challenges and Future Directions

Despite the considerable potential of epigenetic markers in ruminant breeding, several practical and conceptual challenges must be addressed before large-scale implementation becomes feasible. Epigenetic marks differ in their degree of genetic stability, which determines their suitability for long-term breeding. Unlike DNA sequence variants, many epigenetic modifications are partially or extensively reprogrammed during gametogenesis and early embryogenesis, limiting their transgenerational persistence [146]. Genetically regulated DNA methylation sites (e.g., meQTL-associated CpGs) and imprinted regions tend to exhibit relatively greater stability. By contrast, environmentally responsive epigenetic marks are sensitive to external stimuli, while many epigenetic features independently display pronounced tissue specificity and temporal dynamics across developmental stages [147,148]. The practical application of epigenetic information is therefore constrained by environmental sensitivity, developmental specificity, epigenetic reprogramming, genetic-epigenetic interactions, and economic feasibility [149]. Accordingly, epigenetic regulation should not be regarded as an inheritance system equivalent to DNA sequence variation, nor as an independent driver of cumulative long-term genetic gain, but rather as a complementary layer that enhances genomic prediction for low-heritability and environmentally sensitive traits.

From a cost–benefit perspective, although sequencing costs for multi-omics technologies such as WGBS and ChIP-seq are gradually declining, large-scale epigenomic profiling across multiple tissues and time points in breeding populations remains expensive and labor-intensive [150]. Emerging approaches such as enzymatic methyl-sequencing (EM-seq) offer more cost-effective alternatives for population-level studies, yet their routine use in commercial breeding programs still requires economic justification relative to expected genetic gain [151]. From a technical perspective, recent advances in cross-species epigenomic profiling have substantially improved the feasibility of comparative studies in non-model mammals. A mammalian methylation array platform enables robust measurement of DNA methylation at tens of thousands of evolutionarily conserved CpG sites across more than 200 mammalian species, providing a standardized and cost-effective platform for cross-species epigenetic analyses and biomarker development [152].

Technical limitations also remain substantial. Many economically relevant epigenetic markers exhibit strong tissue-, cell type-, and sex-specificity. For instance, methylation profiles differ markedly between reproductive tissues and accessible somatic tissues in cattle, while key regulatory tissues such as the ovary or hypothalamus are difficult to sample in vivo [153]. Moreover, epigenetic data are highly sensitive to developmental stage, environmental exposure, and cell composition. Platform-specific biases and batch effects further complicate data interpretation [154]. A major bottleneck for industry adoption is the lack of standardization. Differences in sampling strategies, laboratory protocols, bioinformatic pipelines, and reporting standards often render datasets poorly comparable across studies. As a result, many datasets function as “data islands,” limiting meta-analyses and the development of broadly applicable prediction models [155]. Establishing standardized guidelines for sampling, data generation, and analysis will be essential for translating epigenetic information into breeding practice.

Epigenetic breeding, while offering opportunities to enhance production efficiency, also raises a range of ethical concerns, particularly regarding animal welfare, the long-term stability of epigenetic states, and broader systemic biological effects. First, animal welfare standards should move beyond the traditional “absence of disease” paradigm toward a framework of quantifiable and validated positive welfare indicators, including musculoskeletal health, cardiovascular function, and behavioral performance, to ensure that technological gains are not achieved at the expense of animal well-being. Second, the potential for transgenerational instability in epigenetic regulation necessitates that breeding strategies incorporate continuous multigenerational epigenetic monitoring and environmental challenge testing to maintain the predictability and uniformity of target traits [139,156]. For example, genes such as *IGF2*, which function within complex regulatory networks, require long-term and systematic evaluation using multi-omics approaches to identify possible metabolic and developmental risks [157]. In addition, the increasing application of epigenomic profiling in livestock breeding raises governance challenges related to data privacy, ownership, and intellectual property. Epigenomic datasets may contain identifiable biological information comparable to genetic data, highlighting the need for robust privacy protection and responsible data-sharing practices [158]. The increasing economic value of genomic and epigenomic innovations introduces intellectual property and ownership challenges that may influence technology dissemination and breeding program competitiveness [159]. Public acceptance represents another critical dimension, as societal perceptions of biotechnology-driven breeding can shape regulatory decisions and adoption pathways [160]. Collectively, these issues underscore the importance of developing harmonized regulatory frameworks and ethical guidelines to support the responsible implementation of epigenetic technologies in livestock production systems.

From a methodological standpoint, incorporating epigenetic markers into genomic prediction frameworks may contribute to improved breeding value estimation by capturing additional sources of phenotypic variation beyond SNP-based models. Multi-omics models such as GOBLUP that integrate methylation data provide opportunities to partition additive genetic and epigenetic components, thereby offering complementary insights into unexplained phenotypic variance and potentially improving prediction accuracy [161]. Looking ahead, epigenetic breeding is likely to shift ruminant breeding from a static genetic paradigm toward a dynamic, multi-layered regulatory framework. Comparative epigenomics across species may reveal evolutionarily conserved regulatory regions relevant to growth, metabolism, and reproduction, supporting cross-species breeding insights [162]. Species-specific differences further complicate the application of epigenetic information in ruminant breeding. Compared with cattle and sheep, epigenetic studies in goats and yak are often constrained by smaller population sizes, limited reference resources, and reduced accessibility of key regulatory tissues, while yak additionally exhibit unique epigenetic adaptations associated with high-altitude and hypoxic environments. Time-series and single-cell multi-omics approaches will further clarify developmental epigenetic reprogramming events and critical windows for intervention [163]. In parallel, artificial intelligence and machine learning are becoming indispensable tools for analyzing high-dimensional epigenetic data, with promising results already reported for predicting feed efficiency and residual feed intake in dairy goats [164].

Overall, while epigenetic technologies are not yet ready to replace conventional genomic selection, they provide a powerful complementary layer of biological information. Taken together, these challenges highlight the key considerations that must be addressed before epigenetic information can be reliably translated into practical ruminant breeding programs (see Box 1 for a concise summary).

Box 1 **Key take-home messages for breeders and researchers.**

Epigenetic markers capture environmentally responsive regulatory variation that is largely independent of DNA sequence variation, providing complementary information to genomic selection, particularly for low-heritability and environmentally sensitive traits (e.g., fertility, feed efficiency, and heat tolerance).

Technical reproducibility remains a major challenge, as epigenetic signals are strongly influenced by tissue type, developmental stage, environmental exposure, and batch effects, underscoring the need for standardized sampling and analytical procedures (e.g., consistent use of milk somatic cells versus blood-derived DNA methylation profiles).

Although sequencing costs are declining, large-scale epigenomic profiling across multiple tissues and time points is still economically restrictive, and cost–benefit evaluations are essential before routine commercial implementation (e.g., comparing whole-genome bisulfite sequencing with targeted methylation panels).

Integrating epigenetic data with genomic and other omics layers can improve trait prediction, but effective data integration is constrained by differences in data scale, temporal resolution, and tissue origin (e.g., combining methylation data with genomic estimated breeding values and transcriptomic profiles).

Species-specific biological characteristics and resource availability must be considered when translating epigenetic findings into breeding practice, as epigenetic architectures and sampling feasibility differ across ruminant species (e.g., differences in follicle biology between sheep wool traits and dairy cattle mammary gland traits).

## 7. Conclusions

**Figure 3:**
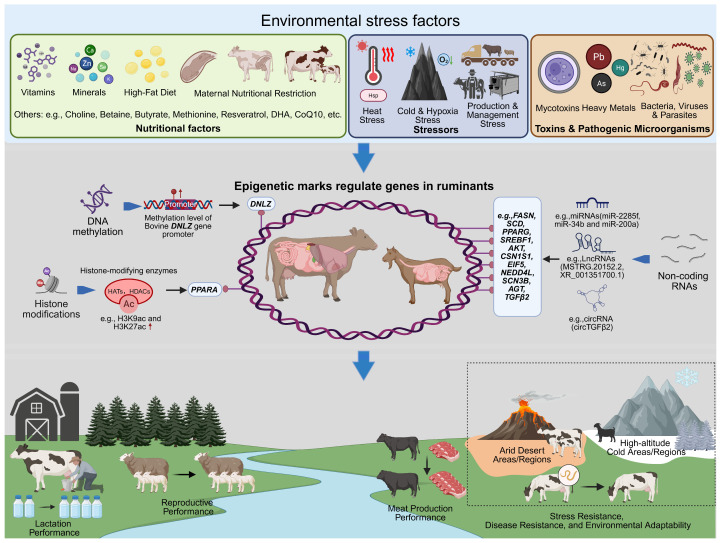
Application of epigenetic regulation in trait formation and breeding of ruminants. Environmental stressors and nutritional factors modulate epigenetic marks in ruminants, including DNA methylation, histone modifications, and non-coding RNAs. These epigenetic mechanisms regulate genes associated with metabolism, lactation, reproduction, and stress and immune responses, thereby influencing economically important traits such as milk yield, meat production, fertility, disease resistance, and environmental adaptability. Epigenetic information can complement genomic selection by improving functional annotation of regulatory regions and enhancing prediction accuracy when integrated with genomic and phenotypic data. Abbreviations: Me, Methylation; Ac, Acetylation; HATs, Histone acetyltransferases; HDACs, Histone deacetylases; H3K9ac, Histone H3 lysine 9 acetylation; H3K27ac, Histone H3 lysine 27 acetylation; miRNAs, MicroRNAs; lncRNAs, Long non-coding RNAs; circRNA, Circular RNA; *PPARA*, Peroxisome proliferator-activated receptor alpha; *PPARG*, Peroxisome proliferator-activated receptor gamma; *SREBF1*, Sterol regulatory element binding transcription factor 1; *FASN*, Fatty acid synthase; *SCD*, Stearoyl-CoA desaturase; *AKT*, Protein kinase B; *CSN1S1*, Casein alpha s1; *EIF5*, Eukaryotic translation initiation factor 5; *NEDD4L*, Neural precursor cell expressed developmentally down-regulated 4-like; *SCN3B*, Sodium voltage-gated channel beta subunit 3; *AGT*, Angiotensinogen; *TGFβ2*, Transforming growth factor beta 2; *DNLZ*, DNL-type zinc finger protein; VA, Vitamin A; VB9, Vitamin B9; VC, Vitamin C; VD, Vitamin D; DHA, Docosahexaenoic acid; CoQ10, Coenzyme Q10; Na, Sodium; K, Potassium; Zn, Zinc; Se, Selenium; Pb, Lead; Hg, Mercury; As, Arsenic; O_2_, Oxygen; Hsp, Heat shock protein. The upward red arrow indicates an increase in the level of epigenetic modification.

Epigenetic regulation provides a critical molecular link between genetic background, environmental exposure, and phenotypic variation in ruminants by modulating gene networks underlying growth, reproduction, immunity, and other economically important traits (Figure 3). Despite substantial progress, current evidence remains largely correlative, and epigenetic signals are strongly influenced by tissue specificity, developmental stage, and environmental context, while standardized protocols and cross-population validation are still limited. Future research should prioritize longitudinal, multi-tissue studies and functional validation to establish causal relationships and improve reproducibility. Although integrating epigenetic information with genomic selection and multi-omics frameworks offers promising opportunities for improving the prediction of low-heritability and environmentally sensitive traits, large-scale implementation in commercial breeding programs will depend on demonstrated cost-effectiveness, methodological harmonization, and responsible governance. At present, epigenetic information should therefore be considered a complementary enhancement to genomic selection rather than a standalone breeding strategy.

## Figures and Tables

**Table 5:** Representative applications of epigenetic marks in ruminant breeding.

Associated Traits	Epigenetic Marks	Application Direction	Key Epigenetic Features	Validation Evidence/Predictive Performance	Readiness Level	References
Fertility and reproductive performance	DNA methylation-based epigenetic biomarkers, ncRNA-mediated regulation	Fertility Prediction, Fertility evaluation and breeding	DMCs/DMRs in fertility-related genes (e.g., *UCHL3*, *KLHL10*, *PLXNB2*, *NPAS1*, *LBX1*, *SORCS2*, *ATG7*, *Peg10*, *Mest*); Differential sperm miRNAs and sncRNAs (e.g., miR-100, miR-29a, miR-449a, miR-1246, oar-miR-200b, oar-miR-370-3p, oar-miR-26b, oar-let-7d) associated with sperm quality and conception rate	Predictive model with independent validation (72% accuracy); additional miRNA associations reported.	Validation	[83,120,123,124,125]
Stress tolerance and disease resistance	Promoter DNA methylation	Stress and disease resilient breeding	CpG methylation changes in stress- and immunity-related genes (e.g., *DNLZ*, *ENOPH1*, *MYL10*, *KIR2DL5A*, *TAAR*)	Association evidence identified; population-level predictive modeling remains limited.	Discovery	[121,126]
Production traits (milk, growth, meat quality)	DNA methylation-based regulation, lncRNA-mediated transcriptional regulation	Improvement of production performance, Enhancement of production traits	Differential DNA methylation in genes related to lactation, growth and muscle development (e.g., *ACTA1*, *MYH11*, *FN1*, *ROCK2*), Differentially expressed lncRNAs associated with mammary gland development and lactation	Exploratory epigenetic associations; no validated population-level prediction model available.	Discovery	[127,128]
Reproductive biotechnology optimization	Histone modification-based regulation	Optimization of assisted reproductive technologies	Histone mark H4K20me3 associated with nuclear transfer embryo development	Functional validation in experimental SCNT systems; not yet translated to breeding-scale applications.	Piloted	[129]
Reproductive performance	DNA methylation-based regulation, ncRNA-mediated regulation	Prolificacy improvement, Improvement of reproductive traits	DMRs in fecundity-related genes (e.g., *SERPINB2*, *NDRG4*, *CFAP43*, *PGF*), Differentially expressed lncRNAs associated with sexual maturation	Candidate DMRs identified; predictive accuracy and large-scale validation not reported.	Discovery	[92,130]

Note: Application maturity ranges from exploratory association studies to independently validated predictive models. Most epigenetic biomarkers remain at the discovery or functional validation stage. Readiness levels: Discovery, early-stage mechanistic or association evidence; Validation, replicated findings with functional or predictive support; Piloted, evaluated in experimental or limited breeding applications.

## Data Availability

No new data were created or analyzed in this study. Data sharing is not applicable to this article.

## Supplementary Materials

The following supporting information can be downloaded at: https://www.mdpi.com/article/10.3390/biology15050416/s1, Figure S1: Integrated layers of epigenetic regulation shaping gene expression; Table S1: Epigenetic regulation of muscle development and adipogenesis in ruminants; Table S2: Epigenetic regulation of reproductive traits in ruminants; Table S3: Epigenetic regulation of metabolism, immunity, and disease in ruminants; Table S4: Epigenetic regulation of ruminant livestock product traits; Table S5: Glossary of key epigenetic terms.

## Abbreviations

The following abbreviations are used in this manuscript:

5caC	5-Carboxylcytosine
5fC	5-Formylcytosine
5hmC	5-Hydroxymethylcytosine
5mC	5-Methylcytosine
Ac	Histone acetylation
AKT	Protein kinase B
ATAC-seq	Assay for transposase-accessible chromatin using sequencing
BLUP	Best linear unbiased prediction
ceRNA	Competing endogenous RNA
ChIP-seq	Chromatin immunoprecipitation sequencing
circRNA	Circular RNA
COBRA	Combined bisulfite restriction analysis
CpG	Cytosine–phosphate–guanine dinucleotide
CTCF	CCCTC-binding factor
ddPCR	Droplet digital polymerase chain reaction
DMC	Differentially methylated cytosine
DMR	Differentially methylated region
DNMT	DNA methyltransferase
DNMT1	DNA methyltransferase 1
DNMT3A/3B	DNA methyltransferase 3A/3B
EWAS	Epigenome-wide association study
EV	Extracellular vesicle
GEBV	Genomic estimated breeding value
GS	Genomic selection
GWAS	Genome-wide association study
G×E	Genotype × environment interaction
HAT	Histone acetyltransferase
HDAC	Histone deacetylase
H3K4me3	Trimethylation of histone H3 lysine 4
H3K9ac	Acetylation of histone H3 lysine 9
H3K27ac	Acetylation of histone H3 lysine 27
H3K27me3	Trimethylation of histone H3 lysine 27
IHC	Immunohistochemistry
lncRNA	Long non-coding RNA
MAPK	Mitogen-activated protein kinas
meQTL	Methylation quantitative trait locus
miRNA	MicroRNA
ncRNA	Non-coding RNA
RRBS	Reduced representation bisulfite sequencing
RNA-seq	RNA sequencing
RT-qPCR	Reverse transcription quantitative polymerase chain reaction
SAM	S-Adenosylmethionine
SCNT	Somatic cell nuclear transfer
scRNA-seq	Single-cell RNA sequencing
snoRNA	Small nucleolar RNA
TAD	Topologically associating domain
TET	Ten-eleven translocation methylcytosine dioxygenase
TF	Transcription factor
WGBS	Whole-genome bisulfite sequencing
WGCNA	Weighted gene co-expression network analysis
