# Supplementary material for: Epigenetic Regulation of Production Traits in Ruminants: Implications for Breeding and Selection
Source: Biology (Basel). 2026 Mar 3;15(5):416. doi: 10.3390/biology15050416 (PMC12984340; doi:10.3390/biology15050416)
Supplement: Supplementary file 1 [file biology-15-00416-s001.zip › biology-4132876-supplementary-Figure S1.pdf]

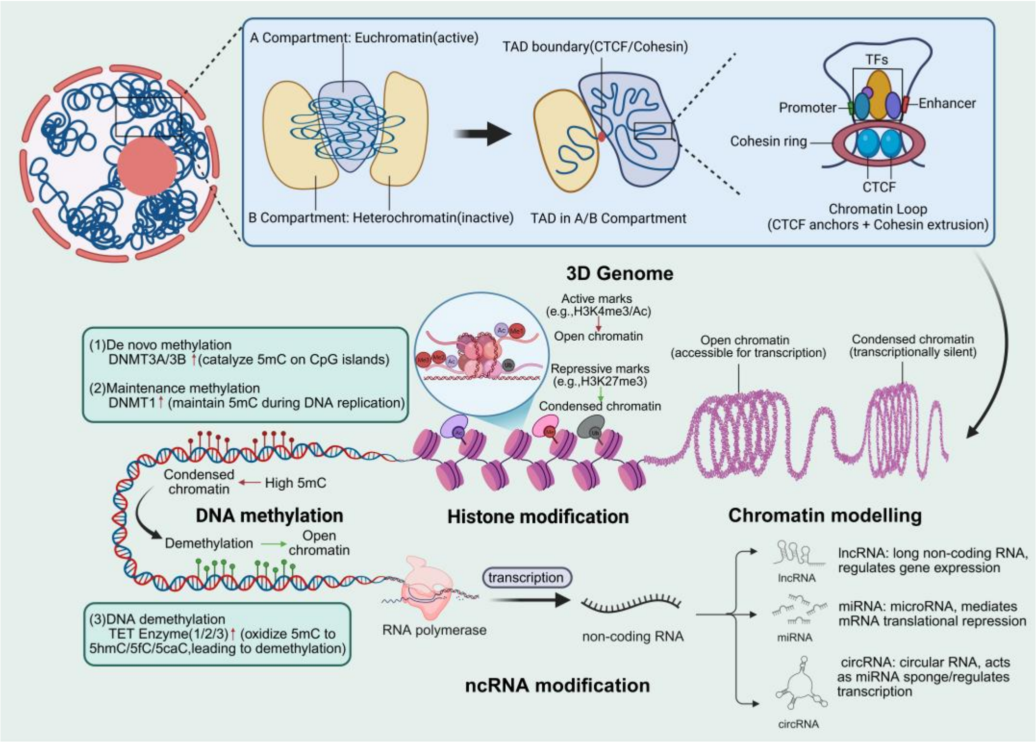

Supplementary Figure S1. Integrated layers of epigenetic regulation shaping gene expression. The diagram summarizes epigenetic regulation across spatial scales. At the 3D genome architecture level, chromosomes segregate into active A (euchromatin) and inactive B (heterochromatin) compartments containing TADs. TAD boundaries are enriched for CTCF and cohesin, which mediate loop extrusion and promoter-enhancer contacts. Chromatin states range from open (transcription-permissive) to condensed (transcriptionally silent). Histone modifications include activating marks such as Ac and H3K4me3, and repressive marks such as H3K27me3, as well as mono-, di-, and tri-methylation (Me1/2/3) and Ub. DNA methylation at CpG sites (5mC) is established by DNMT3A/3B, maintained by DNMT1, and removed via TET1/2/3-mediated oxidation (5hmC, 5fC, 5caC). ncRNAs include lncRNA, miRNA, and circRNA, which regulate transcription and post-transcriptional processes. Together, these layers coordinate gene expression. Abbreviations: TAD, Topologically associating domain; CTCF, CCCTC-binding factor; TFs, Transcription factors; DNMT, DNA methyltransferase; DNMT1, DNA methyltransferase 1; DNMT3A/3B, DNA methyltransferase 3A/3B; TET, Ten-eleven translocation methylcytosine dioxygenase; 5mC, 5-methylcytosine; 5hmC, 5-hydroxymethylcytosine; 5fC, 5-formylcytosine; 5caC, 5-carboxylcytosine; H3K4me3,Trimethylation of histone H3 lysine 4; H3K27me3, Trimethylation of histone H3 lysine 27; Ac, Histone acetylation; Ub, Ubiquitination; ncRNA, Non-coding RNA; lncRNA, Long non-coding RNA; miRNA, MicroRNA; circRNA, Circular RNA.
